# Health-related quality of life in children born with congenital diaphragmatic hernia

**DOI:** 10.1007/s00383-018-4237-1

**Published:** 2018-02-16

**Authors:** Elin Öst, Björn Frenckner, Margret Nisell, Carmen Mesas Burgos, Maria Öjmyr-Joelsson

**Affiliations:** 1 Department of Women’s and Children’s Health, Karolinska Institutet, 171 76 Stockholm, Sweden; 2 Paediatric Surgery Unit, Karolinska University Hospital, Astrid Lindgren Children’s Hospital, 171 76 Stockholm, Sweden; 3 The Red Cross University College, 141 52 Huddinge, Sweden

**Keywords:** Health-related quality of life, Congenital diaphragmatic hernia, Long-term follow-up, Extracorporeal membrane oxygenation.

## Abstract

**Purpose:**

The aim of this study was to examine health-related quality of life (HRQoL) in children born with congenital diaphragmatic hernia (CDH).

**Methods:**

Between 1993 and 2003, a total of 102 children born with CDH were treated at Astrid Lindgren Children’s hospital in Stockholm. In 2012, long-term survivors (*n* = 77) were asked to participate in the present study, which resulted in a 46% (*n* = 35) response rate. The KIDSCREEN-52 questionnaire was used for measuring HRQoL and a detailed review of medical records was performed.

**Results:**

The study participants did not differ from the non-participants in terms of prenatal diagnosis, gender, side of lesion, method of surgical repair, time to intubation, need for ECMO support, or way of discharge from the hospital. Children born with CDH considered themselves to have a good HRQoL, as good as healthy Swedish children. There were only a few significant HRQoL differences within the group of children with CDH, although several median scores in ECMO-treated patients were somewhat lower. Correlations between child and parent scores on HRQoL were low.

**Conclusions:**

Health-related quality of life in children born with CDH is good overall, however, a correlation between the severity of the malformation and HRQoL cannot be excluded.

## Background

Congenital diaphragmatic hernia (CDH) is a rare anomaly with an incidence of approximately 1 per 3000 births [1, 2]. Babies born with CDH most often suffer from acute respiratory distress shortly after being born and immediate intubation is often necessary. The severity of the malformation varies widely and occasionally extra corporeal membrane oxygenation (ECMO) is required. The total length of hospital stay varies from weeks to months and seems to be depending on the size of the diaphragmatic defect at the time for surgical repair [3]. Over the last decade, survival rates have dramatically increased and about two-thirds of all children born with CDH survive to hospital discharge [4] and children, who earlier died of severe CDH, today represent a new group of survivors. Children born with CDH who survive often suffer from morbidities related to pulmonary hypoplasia, pulmonary hypertension, and associated anomalies, but also from the intensive care that these critically ill CDH neonates have been exposed to. Approximately 90% of CDH survivors have some kind of long-lasting associated morbidity [5] where the size of the defect seems to be an isolated indicator for morbidity at discharge from the hospital [3]. Many centres have initiated standardized follow-up programmes to ensure that all morbidity areas are covered [6, 7], including pulmonary-, gastrointestinal-, neurodevelopmental-, and musculoskeletal-related outcomes [6, 8]. Surgical complications in CDH survivors are common and can occur asymptomatically many years after the repair [9, 10]. The early recognition of symptoms may increase survival and prevent secondary morbidity [11].

In recent years, along with an increased survival rate, long-term outcomes in children and adolescents born with CDH have gained more attention, and this is where health-related quality of life (HRQoL) plays a naturally important role [12]. The World Health Organization (WHO) defines quality of life (QoL) as “an individual’s perception of their position in life in the context of the culture and value systems in which they live and in relation to their goals, expectations, standards, and concerns” [13]. Furthermore, WHO states “it is a broad ranging concept affected in a complex way by the person’s physical health, psychological state, personal beliefs, social relationships, and their relationship to salient features of their environment” [13]. In the present study, we used the definition of HRQoL as “a multidimensional construct covering physical, emotional, mental, social, and behavioural components of well-being and function as perceived by patients and/or other observers” [14].

Few centres have published results on HRQoL studies in children born with CDH and, due to discordance in the definitions and measurements of HRQoL, results are difficult to contrast. The aim of this study was to examine health-related quality of life in children born with CDH.

## Methods

### The parents and their children

Astrid Lindgren Children’s hospital is one of four paediatric surgical referral centres in Sweden, and at the time from 1993 to 2003, the only centre with ECMO support available for children. Between the years 1993 and 2003, a total of 102 children with CDH were treated at Astrid Lindgren Children’s hospital, and 84 (82%) of them were discharged alive from our hospital, either to their homes or to a hospital closer to home. The long-term survival rate was, according to the Swedish population register in 2012, 75% (77 children). Similar survival rates have previously been described from the larger cohort where this study population belongs [15]. All 77 children/adolescents with CDH and their parents were asked to participate in this study. In total, 51 families (67%) agreed to participate, five families disagreed and 21 families never answered, despite several invitations. Out of the 51 families who were willing to participate, 35 returned the answered questionnaire (a 46% response rate).

Data on gender, prenatal diagnosis, birth weight, gestational age, side of lesion, method of surgical repair, time to intubation, history of ECMO treatment, and type of discharge from hospital were collected from the case records for all the patients during the time period.

### Questionnaire

KIDSCREEN-52 is a generic questionnaire designed to assess health-related quality of life (HRQoL) in healthy and chronically ill children and adolescents from 8 to 18 years of age [16]. A proxy version is available for parents. The questionnaire is designed to measure children’s and adolescents’ subjective health and well-being, which is the signification of HRQoL [14], and aims to provide a better understanding of perceived health in children and adolescents to identify populations at risk. The KIDSCREEN project was cross-cultural and was developed in 13 European countries, with Sweden as one of the participants. The instrument measures 10 domains on HRQoL distributed over 52 questions: physical well-being (five items); psychological well-being (six items); moods and emotions (seven items); self-perception (five items); autonomy (five items); parent relations and home life (six items); social support and peers (six items); school environment (six items); social acceptance (bullying) (three items); and financial resources (three items) [17]. KIDSCREEN-52 is a valid and reliable questionnaire with Swedish and European normative data available [18]. Answers are given through a five-point scale ranging from never/not at all to always, referring to the previous week and the questionnaire takes about 15–20 min to complete. The proxy version has the same structure as the child and adolescent version, but asks the parent to answer how they think their child feels. The proxy version is a substitute for when a child’s self-report of their HRQoL is not available.

### Ethics

This study was approved by the regional ethical committee in Stockholm, Dnr 2011/472-31/4. Written informed consent was obtained from all adolescents of majority age and the parents to underage children who were included in this study.

### Statistics

A sum score for each of the ten domains was calculated after negatively formulated items were recoded according to standard scoring algorithms. The KIDSCREEN-52 instrument supplies a Rasch model to interpret the results on a standardized interval scale [16]. When transforming the data into the given model, normally distributed *T* values were available. Data are presented as means and SD, maximum, and minimum. Pearson correlation coefficients were used to calculate the correlation between scores of the different domains and age. To measure the correlation between the children’s and parents’ T values, a two-way random single-measure intra-class correlation coefficient (ICC) was used. *p* < 0.05 was considered statistically significant. All statistics were analyzed with the R software program [19].

## Results

### Participant and patient characteristics

The participants did not differ from the entire cohort regarding prenatal diagnosis, gender, side of lesion, method of surgical repair, time to intubation, need for ECMO support, or way of discharge from the hospital. For natural causes, however, there was a difference in survival rates. Among the non-participants, there was no significant difference between the study participants and those who declined or never answered the questionnaire. However, between the group of deceased children and the study´s participants, there were several significant differences. The group of deceased children had a prenatal diagnosis, were intubated within their first 6 h of life, and needed ECMO support more often. For further characteristics, see Table 1. Median age was 13 years and IQR 7 years.

**Table 1:** Demographic data for all children with CDH treated at Astrid Lindgren Children’s hospital 1993–2003 (both children of study participants and non-particpants) *n* (%)

	Entire cohort (*n* = 102)	Study participants (*n* = 35)	Non-participants (*n* = 67)	
			Declined or excluded (*n* = 42)	Deceased (*n* = 25)
Gender				
Male	67 (66)	21 (60)	26 (60)	20 (80)
Female	35 (34)	14 (40)	16 (38)	5 (20)
Prenatal diagnosis	31 (30)	8 (23)	7 (17)	16 (64)*
Birth weight (kg) (mean ± SD)	3.4 ± 0.62	3.5 ± 0.58	3.3 ± 0.66	3.2 ± 0.58
Gestational age (weeks) (mean ± SD)	38 ± 2	39 ± 2	39 ± 3	37 ± 2
Side of lesion				
Left	74 (73)	29 (83)	32 (76)	13 (52)*
Right	14 (14)	4 (11)	6 (14)	4 (16)
Bilateral	1 (1)	0 (0)	0 (0)	1 (4)
Repaired				
Primary	58 (57)	24 (69)	30 (71)	4 (16)
Patch	26 (25)	8 (23)	7 (17)	11 (44)
Intubated within 6 h from birth	73 (72)	21 (60)	28 (67)	24 (96)*
ECMO	29 (28)	6 (17)	6 (14)	17 (68)*
ECMO > once	5 (5)	1 (3)	0 (0)	4 (17)
Referred to other hospital	24 (24)	11 (31)	9 (21)	4 (17)
Survival to discharge	84 (82)*	35 (100)	42 (100)	7 (28)
Long-term survivors (2012)	77 (75)*	35 (100)	42 (100)	0 (0)

*p < 0.05, when compared with study participants

### Results from the KIDSCREEN-52 (long version) questionnaire

**Fig. 1:**
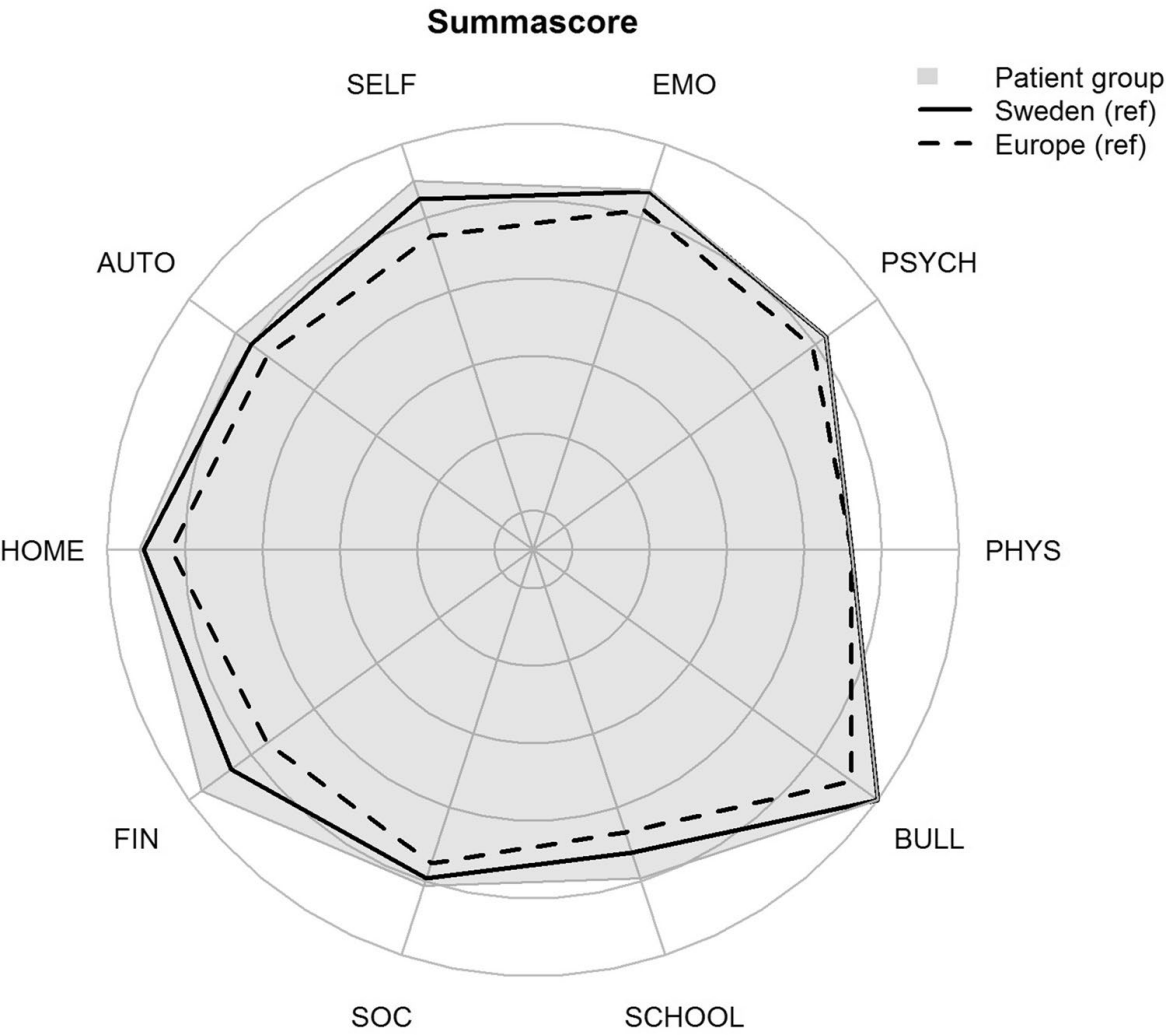
Sum score scale comparison between children with CDH, Swedish- and European normative values. *PHYS* physical well-being, *PSYCH* psychological well-being, *EMO* moods and emotions, *SELF* self-perception, *AUTO* autonomy, *HOME* parent relation and home life, *FIN* financial resources, *SOC* social support and peers, *SCHOOL* school environment, *BULL* bullying

Children born with CDH aged 8–18 years scored higher health-related quality of life compared with European normative data for children of the same age on all domains. Significantly higher scores were found in the study group within the domains self-perception, autonomy, parent relations and home life, financial resources, and school environment (Table 2). When comparing HRQoL in children born with CDH aged 12–18 years and Swedish normative data, similar sum scores were found, except for parent relations and home life scoring significantly higher within the study group (Table 3). Differences between the Swedish and European normative data and the study group are shown in Fig. 1. There were no correlations between the different domains and a child´s age.

**Table 2:** Sum scale T-scores comparison between children with CDH and European normative values, girls and boys 8–18 years

Scale	Children with CDH			Reference		*T* test
	Mean	SD	*n*	Norm-mean	Norm-SD	*p* value
Physical well-being	49.5	8.36	35	50	10	0.728
Psychological well-being	52.4	8.66	35	50	10	0.121
Moods and emotions	53.3	10.81	35	50	10	0.087
Self-perception	56.4	10.76	35	50	10	0.002*
Autonomy	54.0	8.52	35	50	10	0.010*
Parent relation and home life	53.8	9.66	35	50	10	0.030*
Financial resources	55.5	8.72	35	50	10	0.001*
Social support and peers	52.1	9.30	35	50	10	0.192
School environment	54.6	11.29	35	50	10	0.025*
Bullying	52.5	9.03	35	50	10	0.109

*p < 0.05

**Table 3:** Sum scale T-scores comparison between children with CDH and Swedish normative values, girls and boys 12–18 years

Scale	Children with CDH			Reference		*T* test
	Mean	SD	*n*	Norm-mean	Norm-SD	*p* value
Physical well-being	50.5	7.72	23	50.3	7.72	0.472
Psychological well-being	53.5	9.20	23	51.5	9.20	0.171
Moods and emotions	54.3	11.54	23	53.3	11.54	0.327
Self-perception	53.9	10.39	23	53.7	10.39	0.339
Autonomy	55.2	8.95	23	52.7	8.95	0.107
Parent relation ad home life	53.1	10.91	23	53.3	10.91	0.918
Financial resources	56.9	8.59	23	53.4	8.59	0.040
Social support and peers	53.2	9.28	23	52.0	9.28	0.397
School environment	54.5	11.51	23	51.3	11.51	0.211
Bullying	54.2	8.44	23	53.3	8.44	0.323

**Fig. 2:**
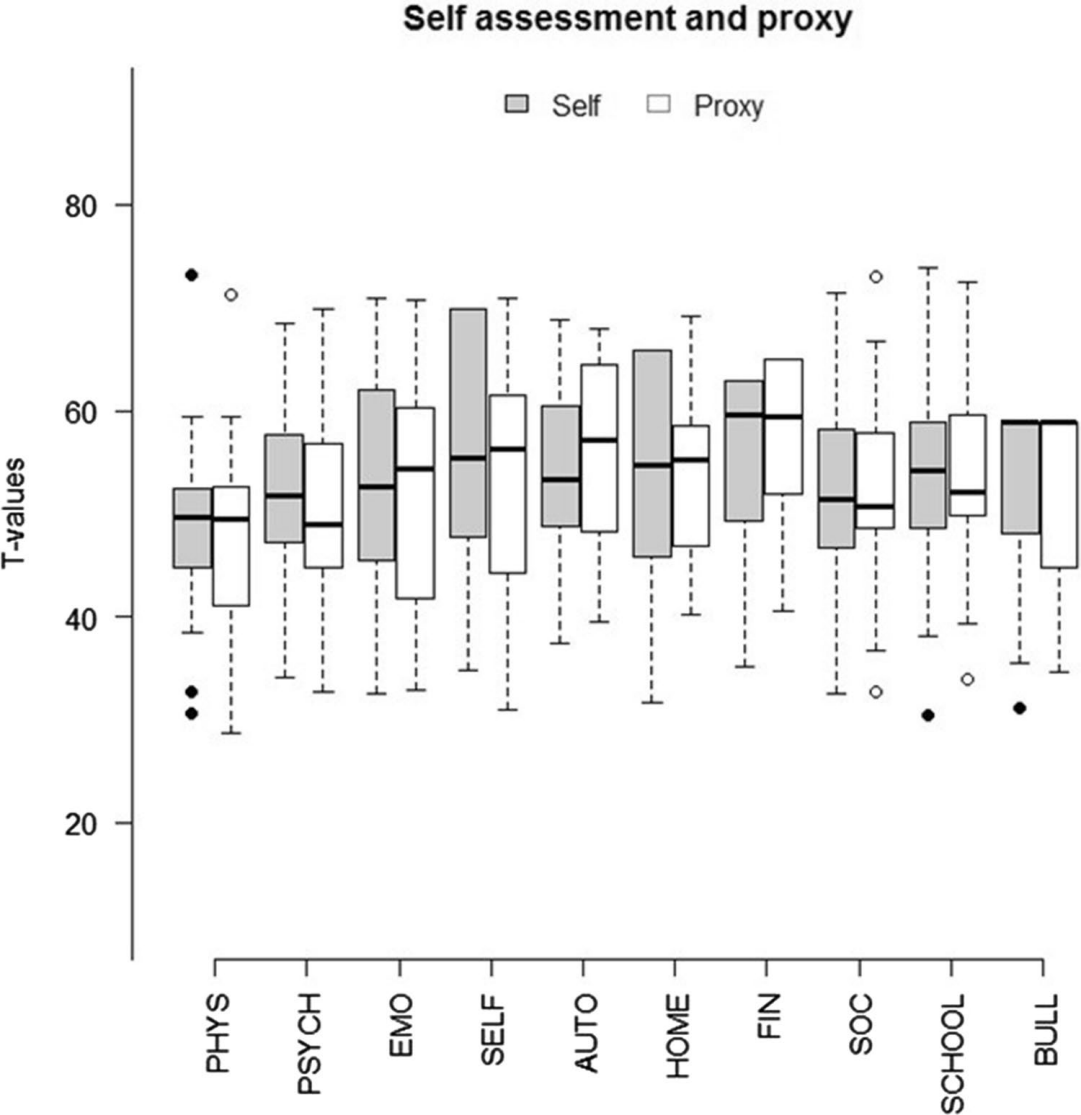
Boxplot comparison between self-assessment (children) and proxy (parent) sum score all scales. *PHYS* physical well-being, *PSYCH* psychological well-being, *EMO* moods and emotions, *SELF* self-perception, *AUTO* autonomy, *HOME* parent relation and home life, *FIN* financial resources, *SOC* social support and peers, *SCHOOL* school environment, *BULL* bullying

Group level scores from parents as a proxy for their children’s HRQoL were similar to the children’s own scores, however, when matching child and parent reports on a pair level, the correlations between scores were low (Table 4; Fig. 2).

**Table 4:** Correlation and comparison between children and parent sum score

	ICC	Lower	Upper	Mean child	Mean parent	*p* value
Physical well-being	0.652	0.380	0.821	49.6	49.5	0.122
Psychological well-being	0.403	0.058	0.663	51.8	48.9	0.364
Moods and emotions	0.376	0.020	0.646	52.7	54.4	0.562
Self-perception	0.537	0.224	0.751	55.4	56.2	0.295
Autonomy	0.548	0.238	0.756	53.2	57.1	0.565
Parent relation and home life	0.731	0.511	0.862	54.6	55.1	0.299
Financial resources	0.388	0.047	0.651	59.6	59.3	0.219
Social support and peers	0.727	0.501	0.860	51.3	50.7	0.666
School environment	0.739	0.519	0.867	54.2	52.1	0.909
Bullying	0.647	0.379	0.815	58.8	58.8	0.489

**Fig. 3:**
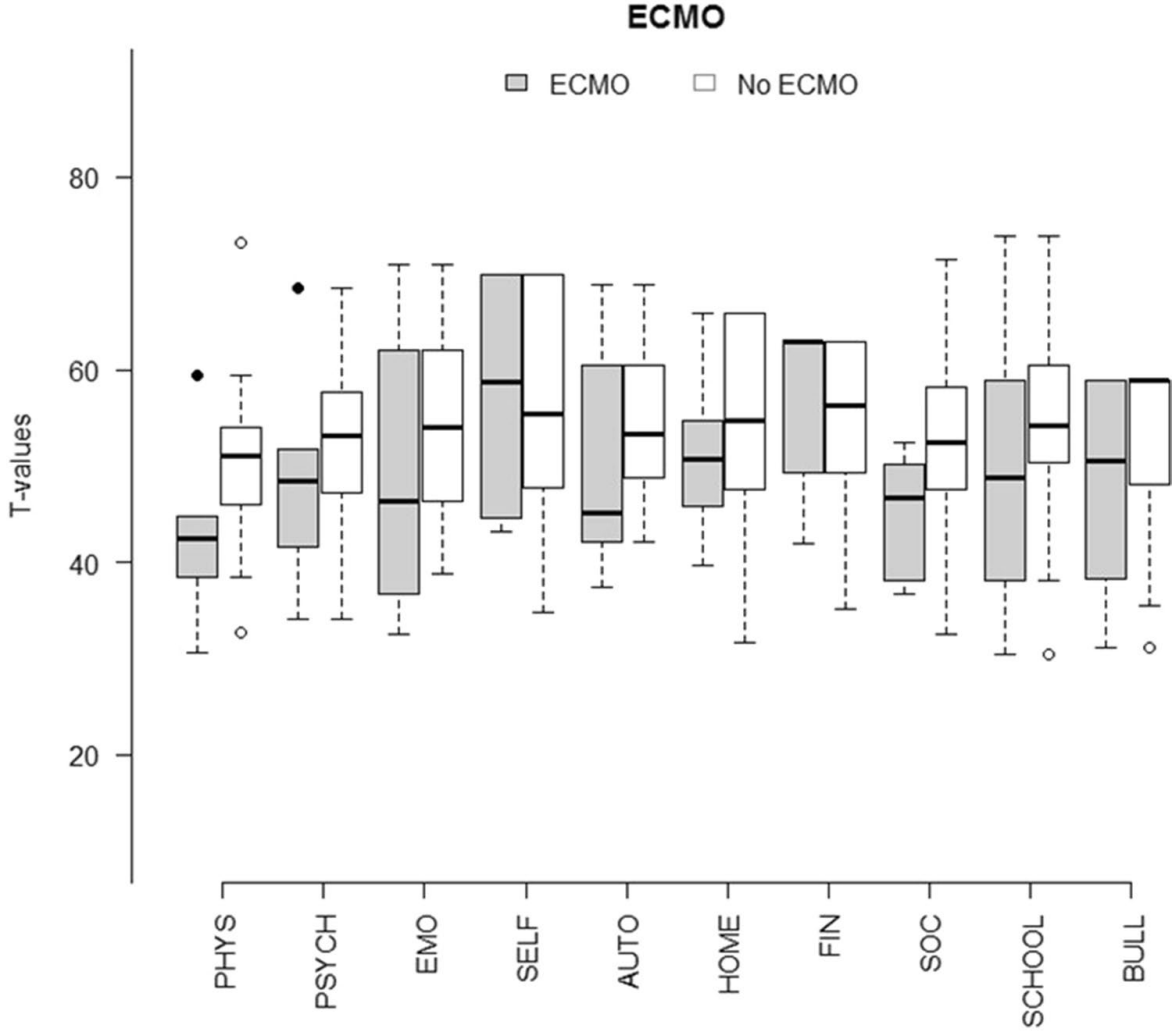
Comparison between sum scores’ all scales according to ECMO support or not. *PHYS* physical well-being, *PSYCH* psychological well-being, *EMO* moods and emotions, *SELF* self-perception, *AUTO* autonomy, *HOME* parent relation and home life, *FIN* financial resources, *SOC* social support and peers, *SCHOOL* school environment, *BULL* bullying

When dividing study participants into subgroups according to the time to intubation, need for ECMO support, side of lesion, and prenatal diagnosis, no significant differences were found, except for social support and peers being negatively affected in the ECMO group. Nevertheless, the median score was lower in children who were in need of ECMO support compared with the others on 8 of the 10 domains, as shown in Fig. 3. For a descriptive summary, please see Table 5.

**Table 5:** Descriptive summary sum score scales’ T-scores divided into subgroups according to severity of the malformation

	Physical well-being			Psychological well-being			Moods and emotions			Self-perception			Autonomy		
	*M*	SD	*N*	*M*	SD	*N*	*M*	SD	*N*	*M*	SD	*N*	*M*	SD	*N*
Time for intubation															
0–6 h	47.7	7.4	20	52.2	8.1	20	53.1	11.5	20	58.3	10.0	20	54.5	9.0	20
>6 h	52.1	9.7	12	53.4	7.5	12	54.7	10.1	12	56.5	11.0	12	55.1	8.0	12
Missing	50.2	7.7	3	49.2	17.5	3	48.8	12.0	3	43.5	7.4	3	46.8	5.8	3
ECMO															
No	50.6	7.6	29	53.1	7.9	29	54.2	9.9	29	56.1	10.4	29	54.9	7.5	29
Yes	43.1	10.6	6	48.8	11.7	6	49.1	14.8	6	57.5	13.6	6	49.8	12.1	6
Side of lesion															
Right	46.1	12.0	4	49.8	14.2	4	48.4	16.4	4	53.2	11.6	4	53.2	11.3	4
Left	49.8	7.9	29	53.2	7.4	29	54.2	9.9	29	57.7	10.3	29	54.0	7.8	29
Missing	52.0	10.3	2	45.9	16.6	2	50.5	16.4	2	43.5	12.2	2	55.4	18.9	2
Prenatal diagnosis															
Yes	49.7	11.0	7	52.4	11.6	7	49.4	13.8	7	56.7	12.9	7	53.5	11.2	7
No	49.4	7.8	28	52.4	8.0	28	54.3	10.0	28	56.3	10.4	28	54.2	7.9	28

	Parent relation and home life			Financial resources			Social support and peers			School environment			Bullying		
	*M*	SD	*N*	*M*	SD	*N*	*M*	SD	*N*	*M*	SD	*N*	*M*	SD	*N*
Time for intubation															
0–6 h	56.0	8.6	20	57.5	7.0	20	52.1	9.4	20	54.6	10.4	20	51.6	10.6	20
>6 h	53.6	9.8	12	54.4	9.2	12	53.0	10.5	12	57.7	11.4	12	54.8	6.2	12
Missing	40.4	5.1	3	47.4	14.1	3	48.4	1.8	3	41.5	9.7	3	49.7	8.4	3
ECMO															
No	54.3	9.9	29	55.1	8.8	29	53.6	9.2	29	55.6	10.3	29	53.5	8.1	29
Yes	51.2	8.9	6	57.1	9.2	6	45.2	6.3	6	49.8	15.5	6	48.0	12.4	6
Side of lesion															
Right	51.1	10.8	4	54.2	5.9	4	46.4	7.9	4	45.1	11.9	4	49.2	13.1	4
Left	54.9	9.3	29	56.1	8.4	29	52.8	9.6	29	56.1	9.4	29	53.0	8.8	29
Missing	42.6	9.8	2	49.0	19.6	2	54.2	5.6	2	52.1	30.6	2	53.5	7.6	2
Prenatal diagnosis															
Yes	59.4	6.9	7	56.4	8.6	7	50.6	12.6	7	51.5	15.9	7	51.0	11.0	7
No	52.3	9.8	28	55.2	8.9	28	52.5	8.5	28	55.4	10.0	28	53.0	8.6	28

*M* mean, *SD* standard deviation, *N* number

## Discussion

The most important result in this study is that children born with CDH express, overall, a high health-related quality of life. Even though the group of children who needed ECMO support scored lower HRQoL on 8 of the 10 domains compared with children without ECMO support, the only significant difference was on the social support and peers domain (Fig. 3).

Extracorporeal membrane oxygenation can be a support for children with CDH to avoid ventilator-induced injury, mainly during the neonatal period when many children with CDH suffer from pulmonary hypertension. On the other hand, ECMO is an invasive and technically challenging treatment associated with serious complications, such as bleeding and thrombosis [20]. The role of ECMO support in CDH remains controversial, even though it is clear that centres that have access to it report the highest survival rates [21]. Nevertheless, the criteria for ECMO treatment are narrow, there has to be an estimated risk of mortality > 80% when the conventional intensive care is applied [20]. In our centre, 28% of the neonates with CDH between 1993 and 2003 met these criteria. The long-term survival rate within this group was low, 41%, and previously published results from our group indicate an increased morbidity within this group of children [22]. We expected HRQoL to be negatively affected in this subgroup of patients treated with ECMO, but despite the visually lower scores of HRQoL, there was no significant difference except for the social support and peers domain (Fig. 3). The results from our study are inline with F. Sheikh et al. where parents of children with CDH as a proxy reported QOL scores similar to parents of healthy children [23]. It should though be mentioned that there is a distinction between the terms QOL and HRQoL, where QOL in general measures subjected well-being and HRQoL is the way that health affects QOL [24]. Furthermore, Sheikh et al. concluded that the parents of children with CDH who had a prenatal diagnosis of the malformation scored good QOL on behalf of their children. Having a prenatal diagnosis of CDH is a predictor of a severe form of the malformation with accompanying higher mortality rates [25]. Similarly, Peetsold et al. demonstrated no correlation between HRQoL and severity of the malformation, with the conclusion that the perception of general health within children with CDH was reduced [26].

In contrast, Michel et al. studied children with CDH born during the same era with preoperative stabilization, gentle ventilation, and access to ECMO with the opposite results. They used KIDSCREEN-27, a shorter form of KIDSCREEN-52, but still with great reliability and found lower HRQoL scores on all the scales compared with norms [27]. Furthermore, they observed an association between low HRQoL, prenatal diagnosis, and length of hospital stay. One possible explanation for this could be that the patient cohorts were not comparable, since the survival rates differ highly between 58% in their study and 82% in our study, and that the rates of ECMO were much lower in their study.

In a report by Koivusalo et al., HRQoL was studied in adults born with congenital diaphragmatic defects, diaphragmatic hernia, and diaphragmatic eventration [28]. These patients were born in the older era when CDH was considered to be an emergency surgical condition and before the introduction of the new successful therapeutical strategies previously stated, including ECMO. In addition, only one patient had patch-repair, representing a less severely affected study population. Furthermore, they reported that most patients had good or satisfactory HRQoL, and found a correlation between lower HRQoL scores and the incidence of gastroesophageal reflux and recurrent intestinal obstruction [28]. Moreover, scar-related problems were brought up as a significant concern with symptoms such as tension and pain, but also as cosmetic issues [28]. A few children/adolescent-mentioned scar issues in free comments in our study, but, at the same time, several questions about self-perception were asked within the KIDSCREEN-52, not confirming any differences in comparison with Swedish norms. Furthermore, Poley et al. examined HRQoL in patients born with CDH aged from 1 to 42 years and found no differences in adolescents and adults over 16 years of age compared with the general population [12]. However, in children 1–4 years of age, they found significantly lower HRQoL on several domains [12]. In the same manner, Peetsold et al. observed differences between the parents’ and children’s scoring of HRQoL, where parents tended to score lower than children [26]. In our study, mean T-scores were similar between parents and children in general, but surprisingly, there was low intra-class correlation (ICC) between the children’s response and their parents on all domains, meaning a low concordance between child and parent agreements. According to the manual for KIDSCREEN, there is a convergent validity between the proxy and child versions, and when both answers are available, the relationship between them can be considered to be valuable information regarding the different points of view [16]. This can however be debated, as Berman et al. recently published their results from a Swedish random population sample where they concluded high child–parent agreement in total [29], but item-by-item child–parent agreement was described as slight to fair in general [29]. Longo et al. recently compared answers from KIDSCREEN-52 in Spanish children with cerebral palsy with their parents and described low correlations between their answers [30]. This clearly shows the difficulties inherent with estimating another person’s HRQoL as the definition is per se subjective and should be measured from the individual’s perspective and, furthermore, cover different health domains, since HRQoL is a multidimensional construct [31]. Being born with a malformation might well provide another view of life and what to be expected, which is hard to understand for anyone else other than the individual in question.

According to other published research on Swedish normative data for KIDSCREEN, Swedish means are known to be higher than European means [16, 29], as in this study. Unfortunately, Swedish normative data for KIDSCREEN-52 are only available for children 12–18 years of age. Berman et al. [29] showed an age difference, where adolescents scored lower well-being than pre-adolescents. In our study, we did not see any age differences; however, our study population was rather small. It is widely known that for many adolescents puberty can be a sensitive time, and there is no reason to believe that anything else would apply for children with CDH.

Here, we observed that children born with CDH experience, overall, good HRQoL. Our institution is a referral centre with long experience of ECMO support in neonates with CDH, and for this reason, this long-term follow-up includes many severely affected children. However, several other reports [26, 27] have shown other results. At the same time, Sheikh et al. recently found indications similar to ours. The large variation between single-institution reports of long-term outcome for children and adolescents born with CDH might be due to patient population and management. We conclude, therefore, that to be able to compare different reports, the patient population must be clearly described, but, most important, the advanced care of children with CDH should be equal for all.

### Strengths and limitations

The strength of this study is that all the patients born between 1993 and 2003 treated for CDH at our hospital with the same standardized postnatal treatment strategy were asked to participate, and the background data for those individuals were available. Due to the Swedish personal identification number (PIN) system, all the children were able to be tracked. However, of the 77 children and parents who were asked to participate in the study, 51 first agreed, but only 35 returned questionnaires. At the same time as the families were asked to fill out the KIDSCREEN-52, we supplied them with another questionnaire, comprising many sensitive questions to be answered, and this might have been a reason for the low number of participants returning the KIDSCREEN-52 questionnaire, which is the main weakness with this study. KIDSCREEN-52 is a commonly used questionnaire with satisfactory psychometric properties [18] with normative data collected on a large number of available controls.

## Conclusion

Children born with CDH seem to experience good HRQoL, as good as healthy Swedish children. The severity of the malformation might impact the experience of HRQoL negatively, yet not significantly.
